# Effect of growth time on Ti-doped ZnO nanorods prepared by low-temperature chemical bath deposition

**DOI:** 10.1016/j.physe.2017.01.009

**Published:** 2017-04

**Authors:** Shaker A. Bidier, M.R. Hashim, Ahmad M. Al-Diabat, M. Bououdina

**Affiliations:** a Institute of Nano-optoelectronics Research & Technology Laboratory (INOR), School of Physics, Universiti Sains Malaysia, USM, 11800 Penang, Malaysia; b Department of Physics, College of Science, University of Bahrain, PO Box 32038, Bahrain

**Keywords:** Ti-doped ZnO nanorod, Growth Time, EFTEM, Chemical Bath Deposition

## Abstract

Ti-doped ZnO nanorod arrays were grown onto Si substrate using chemical bath deposition (CBD) method at 93 °C. To investigate the effect of time deposition on the morphological, and structural properties, four Ti-doped ZnO samples were prepared at various deposition periods of time (2, 3.5, 5, and 6.5 h). FESEM images displayed high-quality and uniform nanorods with a mean length strongly dependent upon deposition time; i.e. it increases for prolonged growth time. Additionally, EFTEM images reveal a strong erosion on the lateral side for the sample prepared for 6.5 h as compared to 5 h. This might be attributed to the dissolution reaction of ZnO with for prolonged growth time. XRD analysis confirms the formation of a hexagonal wurtzite-type structure for all samples with a preferred growth orientation along the c-axis direction. The (100) peak intensity was enhanced and then quenched, which might be the result of an erosion on the lateral side of nanorods as seen in EFTEM. This study confirms the important role of growth time on the morphological features of Ti-doped ZnO nanorods prepared using CBD.

## Highlights

• Ti-doped ZnO nanorods have been prepared using chemical bath deposition method on silicon substrates.
• Effect of immersing time on the morphological and structural properties was investigated.
• FESEM images show that the morphological properties were strongly affected by increase the growth time.
• EFTEM images reveal strong erosion on the lateral direction while the axial direction is enhanced.
• XRD patterns show that the effect of growth time in reduce the (100) direction and enhance the (002).

## Graphical abstract

EFTEM images of (a) sample prepared at 5 h; and (b) sample prepared at 6.5 h.

Increase the growth time causes an erosion in lateral side -(100) direction XRD- and enhances the axial direction -(002), XRD.

**Figure:**
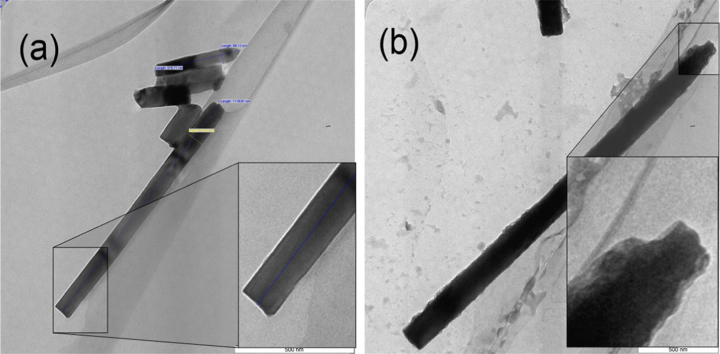

## 1 Introduction

Zinc oxide (ZnO) has gained a particular interest among other metal semiconductor oxides due to its outstanding and unique properties including its direct-wide band gap (3.37 eV), large binding energy (60 eV) at room temperature [1], low-cost material [2], nontoxic and chemically-inert material [3]. ZnO was employed in various fields like light-emitting diodes, UV-photo-detectors, solar cells, gas sensors, pH sensors, and nano-lasers [4], [5].

It is well-known that doping treatment is usually initiated to induce structural and microstructure modifications resulting in the improvement of materials properties thereby higher performance for potential applications [6], [7]. It has been reported that ZnO was doped by various metals such as Al [8], Cu [9]. Ga [10], etc. However, titanium (Ti) was found to be a promising candidate for ZnO doping because its ionic size (Ti^+4^) is smaller than that of Zn^+2^ [11], which facilitates its incorporation within ZnO crystal lattice [7] without any structural alteration or creation of microstructural defects, according to Hume-Rothery rules (ionic radius difference less than 15% for the formation of solid solution). In addition, Ti has a higher valence electrons when compared to Zn, hence its dissolution into ZnO host lattice may reduce the electrical resistivity and improve the electrical properties of ZnO by providing additional free electrons [12], [13].

Several techniques have been proposed for the preparation of Ti-doped ZnO (TZN) thin films such as radio frequency (RF) sputtering [14], pulsed laser deposition (PLD) [15], chemical vapor deposition (CVD) [16], and atomic layer deposition (ALD) [17]. These methods are costly and need high-temperature and/or high vacuum ambient, especially when compared to chemical bath deposition method (CBD). The CBD method possesses many advantages such as low cost, no sophisticated procedures and equipment, and low working temperature [18]. It has been reported that CBD parameters can be easily controlled, and optimized to prepare nanostructures with desirable morphology and properties; whereas the growth time has been considered as an important key factor.

To the best knowledge of the authors, Ti-doped ZnO nanostructures(TZN) prepared using CBD have been rarely reported in the literature. In fact, the effect of growth time on the evolution of structure, microstructure and properties is reported for first time in this paper, It is expected that this study will significantly deliver interesting results and promising nanostructures with unique properties offering potential applications in particular for optoelectronic devices.

In this research work, TiO_2_ is used as a source of doping ZnO. However, its reaction with ZnO is unfeasible at room temperature due to its insoluble nature [19]. For that reason, TiO_2_ was firstly pre-treated in 40 kHz ultrasonic bath at a temperature considerably above ambient temperature. This treatment will generate extraordinary conditions that involves high temperature of about 5000 K and high pressure of about 1000 atmospheric pressure [20]. These conditions play a key role in the rupture of TiO_2_ molecules leading to the formation of Ti^+4^, which will dissolve within ZnO host lattice. The Ti-doped ZnO nanostructures are grown onto Si substrate using chemical bath deposition method. The as-fabricated films are then carefully examined.

## 2 Experimental part

### 2.1 Fabrication of Ti-doped ZnO nanostructures

A p-type silicon substrate (111) was cut into small pieces with 1 cm×1 cm dimensions. The pieces were subjected to cleaning procedures: (i) firstly by dipping into H_2_O:H_2_O_2_:NH_4_OH (5:1:1) solution at 70 °C for 10 min; (ii) then rinsing in deionized water (DI); (iii) after that, the substrates were dipped into a solution containing H_2_O:HF (50:1) at 70 °C for 1 min followed by rinsing with DI; (vi) and finally, dipping in H_2_O:H_2_O_2_:HCl (6:1:1) solution at 70 °C for 10 min, removed, rinsed and washed using DI, and then dried using nitrogen gas. After that, the substrates were seeded with 50 nm of ZnO using a very high purity ZnO target (99.999%) using RF system (HHV- Auto500) at room temperature in Ar gas ambient with a working power and pressure of 150 W and 5.6 mTorr, respectively.

One beaker containing 20 mM of zinc nitrate hexahydrate Zn(NO_3_)_2_·6H_2_O (Sigma Aldrich) with an equal molar concentration of hexamethylenetetramine (C_6_H_12_N_4_) (Sigma Aldrich), was dissolved in 80 ml of DI. The TiO_2_ solution used to prepare TZN samples, was prepared by adding 0.4 g of TiO_2_ (Anatase, Sigma Aldrich) into 40 ml ethanol (99.97%), and then pre-treated using an ultrasonic system (Branson 1510–40 kHz) for 2 h. The deposition reaction was prepared by adding 5 ml of TiO_2_ solution. The prepared samples of TZN-2, TZN-3.5, TZN-5, and TZN-6.5 were synthesized at different deposition time (2, 3.5, 5, and 6.5 h, respectively). The seeded substrates were vertically suspended in the beaker containing the solutions. The beaker was subsequently placed in a furnace oven at 93 °C. The substrates were removed after different periods of time: 2, 3.5, 5, and 6.5 h, and washed in de-ionized water (DI) for 15 min, and then dried with nitrogen gas. Finally, the as-grown samples were kept at room temperature for 24 h before further characterizations.

### 2.2 Characterization techniques

The morphological features of the as-prepared samples were investigated using field emission scanning electron microscopy (FESEM) integrated with energy dispersive spectroscopy (EDS) FEI Nova NanoSEM 450 system. Also, Energy field Transition Electron Mirscope (EFTEM) ZEISS LIRA120 system. The structural characterization was performed using high-resolution X-ray diffraction using HR-XRDPANalyticalX’Pert Pro MRD system equipped with Cu-Kα radiation source (λ_Cu_=0.1541 nm) operating at 40 kV and 20 mA. Fourier transform infrared spectroscopy (FTIR) measurements were carried out using Perkin Elmer Spectrum GX with a mid-infrared area range 370–4000 cm^−1^.

## 3 Results and discussion

### 3.1 FESEM observations

**Fig. 1:**
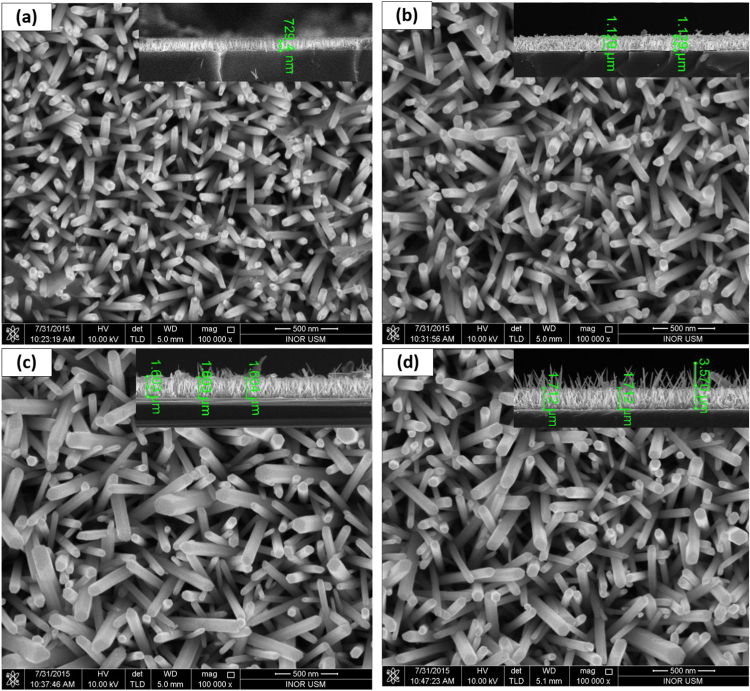
FESEM images of (a) TZ-2; (b) TZ-3.5; and (c) TZ-5; and (d)TZ-6.5.

Fig. 1 shows FESEM images of TZN films grown onto Si substrates by ultrasonic integrated chemical bath deposition (US-CBD) at 93ᵒC. It can be seen that all films show high-quality nanorods (NRs) grown vertically along c-axis direction (perpendicular to the surface of Si substrate). The morphological parameters including the average diameter and length are investigated. From the cross-sectional images (Fig. 1), it can be observed that the length of the as-grown NRs strongly increases as the deposition time increases. The mean length of 729 nn for TZN-2 increases sharply to reach 1129 nm for TZN-3.5. For much longer deposition time, the length of NRs continues to increase to 1604 nm for TZN-5 then 1712 nm for TZN-6.5. It can be noticed that the length-grown rate (length/time) strongly drops from (365 nm/h) for TZN-2 for 2 h compared to (263 nm/h) for 6.5 h. This behaviour may be due to the consumption of (Zn^+2^, OH^−1^) with time; similar results have been previously reported in the literature [21].

On the other side, the average diameter of the as-grown NRs is found to slightly increase as the growth time increases: 46, 55, 75 and 82 nm for TZN-2, TZN-3.5, TZN-5, and TZN-6.5, respectively. The growth-rate of diameter (diameter/time) can be estimated as 23, 15.7, 15, and 12.6 nm/h for TZN-2, TZN-3.5, TZN-5, and TZN-6.5, respectively. Interestingly, these results indicate a sharp reduction in the diameter growth-rate as the deposition time increases, which may be associated with the reduction in the concentration of (Zn^+2^, OH^−1^) species as the deposition time increases. The morphological parameter listed in Table 1.

**Fig. 2:**
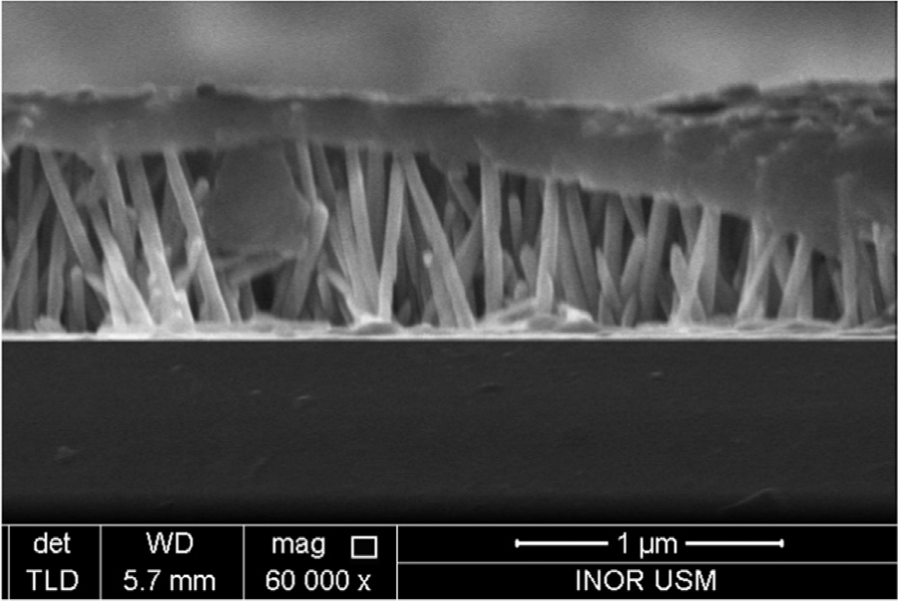
FESEM images of TZ-2-surface layer.

A thin layer of nanoparticles (NPs) can be observed at the surface of the TZN-2 sample, see Fig. 2. This layer serves as a supplier of ZnO NPs, which are formed during the first stage of the chemical reaction, and then NRs grow-up based on this seed layer. The as-formed ZnO NPs seed layer can be considered as a stand for the growth of NRs [22].

Moreover, with increasing the deposition time, it is noticed that the NRs grow preferentially along the axial direction. The axial growth rate is found more pronounced than the lateral growth rate, which can be attributed to low surface free energy within c-axis [23], [24]. Hexamethylenetetramine also plays a crucial role in promoting the growth of NRs into c-axis [21]. So, the synthesis of ZnO via chemical reaction with HMT, in general, produces well-aligned NRs. After 3.5 h of deposition (TZN-3.5), it can be noticed that the NRs length increases without the pre-formation of NPs thin layer, as shown in Fig. 2. This may be due to the consumption of the pre-formed NPs for the growth of NRs. The TZN-5 sample shows much more NRs are horizontally distributed in their upper surfaces. However, the TZN-6.5 sample has no extra NRs in their surface, as well as, some NRs are found lengthy; i.e. about 3750 nm. This behaviour occurring with the increase in the immersion during deposition process may be attributed to the reverse reaction consisting in the dissolution of horizontally-formed NRs for TZN-5 $(\mathrm{ZnO}{\to \mathrm{Zn}}^{+2}+2{\mathrm{OH}}^{-1})$ [22]. The resultant ions (Zn^+2^, OH^−1^) are used to promote the growth of NRs along c-axis direction. As a result, the length of NRs will be increased. Also, it can be noticed that the diameter of the lengthy NRs (3750 nm) is much less than the normal one (1712 nm), and this may be attributed to slow lateral growth rate with the increase of reaction time, where the concentration of (Zn^+2^, OH^−1^) is reduced. In addition, the dissolution reaction along the lateral side is found more significant than that along axial direction, which also contributed in the formation of thinner NRs with increasing the immersion time [25].

### 3.2 EFTEM observations

**Fig. 3:**
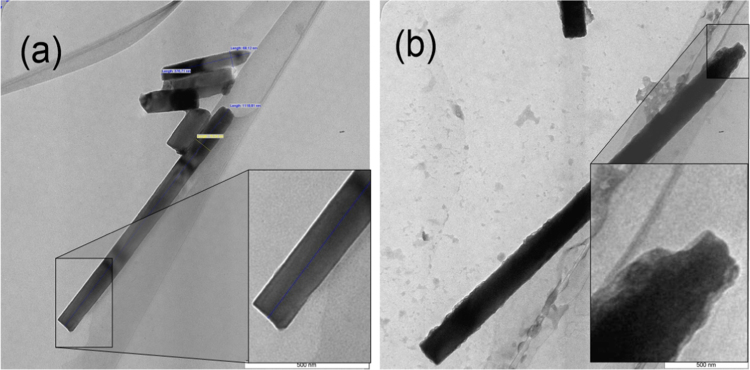
EFTEM images of (a) TZ-5; and (b) TZ-6.5.

Typical EFTEM images of TZN-5 and TZN-6.5 are shown in Fig. 3. It can be seen that NRs in both samples were grown into c-axis direction. For TZN-5 (Fig. 3(a)), the NRs have a uniform diameter; i.e. around 70 nm, no worthy erosion and sharp tip can be easily detected on their surfaces. This may be referred to the deposition reaction of ZnO lattice structure, with no dissolution reaction. However, for TZN-6.5 (Fig. 3(b)), the NRs have sharp tip and a clear erosion over the entire surface, especially in the tip. The inset exhibits strong erosion in lateral sides of the tip with growth into axial direction. The erosion in lateral of the tip improves the growth into axial direction, so; NRs have extra-length as shown in FESEM image. The lateral area is more than the axial area, so, the dissolution is more pronounced along the lateral sides as compared to the axial side. This dissolution would increase the concentration of Zn^+2^ and OH^−1^ ions, which may promote the growth along the c-axis direction. It can be highlighted that EFTEM observations are in good agreement with FESEM. The remarkable difference between TZN-5 and TZN-6.5 as shown after 1.5 h, indicates the importance effect of growth time on the morphological features of the as-prepared NRs. Similar results have been reported in the literature; the erosion was done after 10 h [26].

### 3.3 XRD analysis

**Fig. 4:**
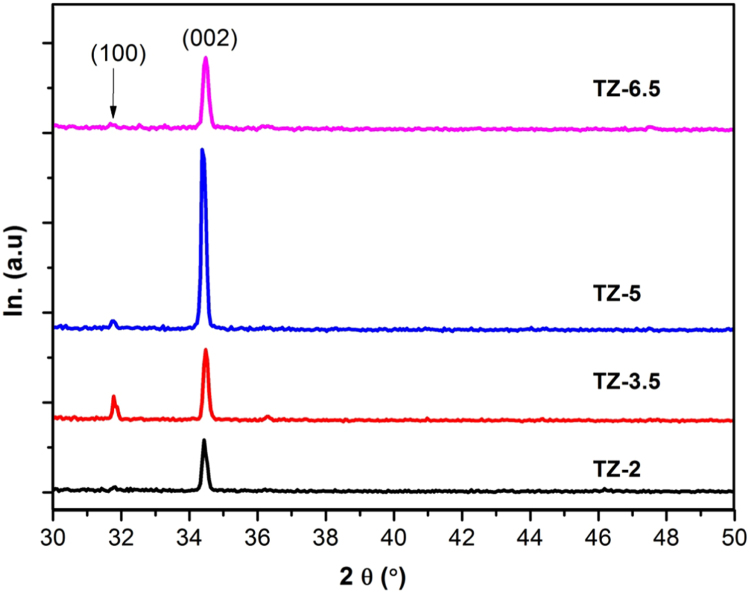
XRD patterns of Ti-doped ZnO films repared at various growth times.

XRD patterns for the as-grown TZN NRs are shown in Fig. 4. All TZN samples revealed wurtzite hexagonal structure of ZnO phase in good agreement with JCPDS card No. (01-080-0074). for all films, the (002) peak is the most intense, which confirms the preferred growth along c-axis direction. No additional minor peaks related to TiO_2_ or other phases can be detected within the limit of resolution of X-ray diffractometer; this favours the possible dissolution of Ti ions (after complete decomposition of TiO_2_) within ZnO crystal lattice [27]. Ti^+4^ has smaller ionic radius (0.068 nm) compared to that of Zn^+2^ (0.074 nm), so Ti^+4^ can easily substitute Zn^+2^ sites and/or occupy interstitial sites within ZnO host lattice [28].

From XRD results, it can be seen that the intensity of (002) peak gradually increases as the growth time increases til 5 h, and then decrease slightly. On the other hand, the intensity of (100) peak has strongly increased for TZN-3.5 and then decreases for TZN-5, then drops drastically for 6.5 h for TZN-6.5. Since (002) and (100) peaks reflect the axial and lateral directions, respectively, hence an increase in the intensity of (002) indicates a preferential growth along c-axis, while a decrease in the intensity of (100) denotes a dissolution in lateral side. These results are once again in good agreement with both FESEM and FETEM obervations as mentioned in previous sections.

The crystallite size D, lattice constant “c”, and strain (εzz) of the as-grown films were calculated for (002) reflection using Scherer formula [29] Eq. (1), Eq. (2), and Eq. (3) respectively:

(1) D=0.9λ∕βcosθ………

(2) c=λ∕sinθ…………….…

(3) $$\varepsilon \mathrm{zz}=\frac{c-c_{0}}{c_{0}}\times 100\%\ldots \ldots$$

where λ is the wavelength of the X-ray radiation source which equal to (0.15406 Å), β and θ represent the full width at half maximum (FWHM) and the diffraction angle of (002) reflection, respectively. c_0_ is the lattice constant of unstrain ZnO structure which equal to (0.52125 nm). The lattice parameters were listed in Table 2.

A clear decrease in the FWHM with increase the reaction time can be noticed; which indicated to improve the crystalline quality. However, the sample prepared at 6.5 h have large FWHM, this may be due dessolution reaction. In this reaction the ZnO lattice converted to solved ions (Zn^+2^, and OH^−1^). This result is in agreement with both FESEM and HFTEM were mentioned above. The crystallite size was significantly increased with prolong the reaction time til 5 h, after that, it was sharbly decreased. Which also confirmed the erosion in the as prepared nanostructure. The lattice constant was reducesed with as increase the immersing time. It can be suggested that, in first time, the concentration of ions (Zn^+2^, OH^−1^ and then Zn^+2^, O^−2^) is high, which allow to them (Zn^+2^, O^−2^) to come into lattice structure, which increases the interstitial defects (Zn, and O) within lattice structure, that could increase the lattice constant as in first sample [30], with increase the time, these ions might structure into the normal sites and/or come out to the reaction solution. The restructuring of interstitial defects (Zn, and O) may reduce the vacancies (Zn, and O) which increases the crystallite size. As will as reduces the interstitial defects may decrease the lattice constant.

From the Table 2 it can be seen that all prepared samples have a compressive strain. Increase the reaction time increased the compressive strain in the lattice structure. This behaviour could refer to reduce the interstial defects (Zn, O) within lattice structure as mentioned in above.

It can be noted that the sample was prepared at 5 h showed the maximum (002) intensity, minimum FWHM and large crystallite size. Which means this time is the optimum deposition time to get high quality Ti-doped ZnO nanorods using low cost CBD.

### 3.4 FTIR spectroscopy

**Fig. 5:**
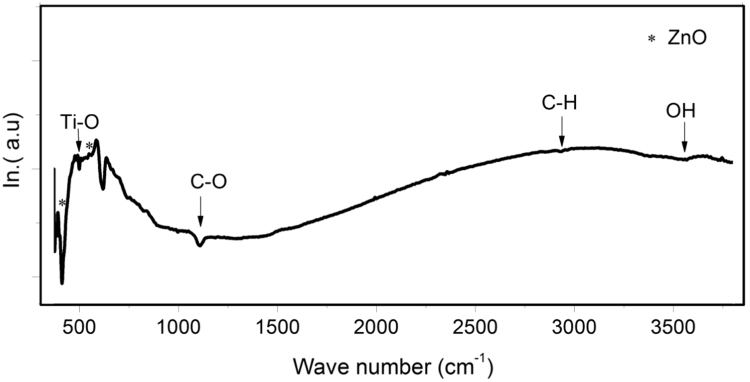
FTIR spectrum of Ti-doped ZnO film.

FTIR is a useful technique used to explore the chemical bonds. Fig. 4 shows the evolution of FTIR spectra recorded in the diffuse reflectance mode of TiO_2_-doped ZnO NRs (TZN) measured in the wavenumber region 370–4000 cm^−1^. The oservered shoulder at 500 cm^−1^ is certified to Ti-O bond [31], hence once again confirm that TiO_2_ decomposes followed by the dissolution of Ti^4+^ ions within ZnO host lattice by occupying most probably Zn^2+^ sites. No additional bonds can be detected, which confirms that the as-grown Ti-doped ZnO NRs are single phase (Fig. 5).

## 4 Conclusion

In this study, Ti-doped ZnO nanorod-arrays are effectively grown onto Si substrates using a novel cost-effective method that consists of chemical bath deposition (CBD) method at 93 °C. FESEM obervations reveal high-quality nanorod-arrays. The morphological parameters (length and diameter) are dramatically changed with prolonged immersion duration time. This may be due to the fast growth rate towards c-axis direction and slow lateral rate. EFTEM obervations show a strong erosion along lateral sides in particular for the sample deposited at longer time (6.5 h). XRD analysis confirms the hexagonal wurtzite structure for all Ti doping concentrations. The intensity of (002) peak increases while of the lateral direction (100) increases then decreases to finally drops abruptly. This behaviour could be due to the fast growth-rate along c-axis and the dissolution reaction along the lateral sides. FTIR spectroscopy analysis confirms the incorporation of Ti within ZnO crystal lattice. This study reports the important role of the deposition time on morphological and structural characteristics of Ti-doped ZnO (TZN) NRs. This will play a key role for potential application such as UV-, gases-, and bio-sensors.

## Figures and Tables

**Table 1:** The morphological parameters of Ti-doped ZnO samples.

Films	Time-h	Length-nm	Length growth rate=Length/time, nm/h	Diameter-nm	Diameter growth rate=diameter/time, nm/h
TZN-2	2	729	364.5	46	23
TZN-3.5	3.5	1129	322.6	55	15.7
TZN-5	5	1603	320.6	75	15.0
TZN-6.5	6.5	1712	263.4	82	12.6

**Table 2:** (002) peak position, FWHM, crystallite size, lattice parameter “c”, and strain as calculated from XRD.

**Films**	**Growth time-h**	**FWHM (°)**	**D (nm)**	**c (nm)**	**Strain (%)**
TZN-2	2	0.170	44.7	0.52031	−0.180
TZN-3.5	3.5	0.165	46.1	0.51977	−0.284
TZN-5	5	0.158	48.2	0.52002	−0.236
TZN-6.5	6.5	0.190	39.9	0.51981	−0.275
